# Augmented Growth Hormone Secretion and Stat3 Phosphorylation in an Aryl Hydrocarbon Receptor Interacting Protein (AIP)-Disrupted Somatotroph Cell Line

**DOI:** 10.1371/journal.pone.0164131

**Published:** 2016-10-05

**Authors:** Takashi Fukuda, Tomoko Tanaka, Yuriko Hamaguchi, Takako Kawanami, Takashi Nomiyama, Toshihiko Yanase

**Affiliations:** 1 Department of Endocrinology and Diabetes Mellitus, Faculty of Medicine, Fukuoka University, Fukuoka, Japan; 2 Department of Bioregulatory Science of Life-related Diseases, Faculty of Medicine, Fukuoka University, Fukuoka, Japan; University of Michigan, UNITED STATES

## Abstract

Aryl hydrocarbon receptor interacting protein (*AIP*) is thought to be a tumor suppressor gene, as indicated by a mutational analysis of pituitary somatotroph adenomas. However, the physiological significance of *AIP* inactivation in somatotroph cells remains unclear. Using CRISPR/Cas9, we identified a GH3 cell clone (termed GH3-FTY) in which *Aip* was genetically disrupted, and subsequently investigated its character with respect to growth hormone (Gh) synthesis and proliferation. Compared with GH3, GH3-FTY cells showed remarkably increased Gh production and a slight increase in cell proliferation. Gh-induced Stat3 phosphorylation is known to be a mechanism of Gh oversecretion in GH3. Interestingly, phosphorylated-Stat3 expression in GH3-FTY cells was increased more compared with GH3 cells, suggesting a stronger drive for this mechanism in GH3-FTY. The phenotypes of GH3-FTY concerning Gh overproduction, cell proliferation, and increased Stat3 phosphorylation were significantly reversed by the exogenous expression of *Aip*. GH3-FTY cells were less sensitive to somatostatin than GH3 cells in the suppression of cell proliferation, which might be associated with the reduced expression of somatostatin receptor type 2. GH3-FTY xenografts in BALB/c nude mice (GH3-FTY mice) formed more mitotic somatotroph tumors than GH3 xenografts (GH3 mice), as also evidenced by increased Ki67 scores. GH3-FTY mice were also much larger and had significantly higher plasma Gh levels than GH3 mice. Furthermore, GH3-FTY mice showed relative insulin resistance compared with GH3 mice. In conclusion, we established a somatotroph cell line, GH3-FTY, which possessed prominent Gh secretion and mitotic features associated with the disruption of *Aip*.

## Introduction

Germline mutations in the aryl hydrocarbon receptor interacting protein gene (*AIP*) predispose to pituitary adenomas, mainly somatotropinomas. These *AIP* germline mutations have been identified in 15%–20% of patients with familial isolated pituitary adenoma (FIPA) and in 3%–5% of patients with sporadic pituitary adenomas [1–5]. The prevalence of these mutations rises to 40%–50% in families with familial acromegaly and families with prolactinomas or somatotropinomas [2, 4], and to 10%–15% even in sporadic cases of prolactinomas or somatotropinomas [6].

AIP demonstrates strong amino acid sequence homology between rats and mice, rats and humans, and mice and humans at 97.0%, 94.0%, and 94.2%, respectively, indicating that it is highly conserved between species. Most common *AIP* alterations result in amino acid substitutions or a truncated AIP protein particularly within the C-terminal, which contains three tetratricopeptide repeats (TPR) responsible for protein–protein interactions [3, 7] Such tumors containing *AIP* mutations typically have a tendency to occur in individuals at a younger age, to become larger and more aggressive [1–6], and to be resistant to somatostatin analogs which are the first-line drug therapy for acromegaly [3, 4, 8, 9]. *AIP* has been postulated to be a tumor suppressor gene from several experimental findings about its *in vitro* function. These include, an *in vitro* culture experiment using a forced expression system which revealed that wild-type AIP suppresses cell proliferation whereas mutant AIP loses this effect, and that partial knockdown of *AIP* by small interfering RNA (siRNA) leads to increased cell proliferation [3, 10–13].

While the molecular mechanisms of pituitary tumorigenesis by *AIP* inactivation remain unclear, several mechanisms have been proposed; AIP inactivation results in a failure to inhibit cyclic adenosine monophosphate (cAMP) production through dysfunctional G-protein alpha-i signaling [13], while *AIP* mutations disturb the interaction with phosphodiesterases, thus leading to an increase in cAMP production [11]. With respect to the relatively insensitive response of some somatotropinomas to somatostatin analogs, the decreased changes in expression of the antiproliferative gene zinc-finger regulator of apoptosis and cell-cycle arrest (ZAC-1; also known as *PLAGL1*) by *AIP* inactivation has been suggested to be a mechanism [14, 15]. *ZAC1* may exert an antiproliferative effect by inducing apoptosis and G1 cell cycle arrest [16].

The above hypothesis of AIP action is mostly based on clinical observations combined with mutational analysis, immunohistochemical studies of pituitary tumors, and *in vitro* experiments using exogenous expression of wild-type or mutant *AIP* in pituitary cells or siRNA knockdown of *AIP*, particularly in rat pituitary GH3 cells. However, the phenotype of complete knockdown of *AIP* in GH-producing cells has not been clarified. In *Aip* knockout mice, heterozygous mice were extremely prone to pituitary adenomas, whereas the total lack of *Aip* resulted in embryonic lethality [17].

A rat pituitary tumor cell line, GH3, was first described as a homogenous clonal cell line that secretes Gh [18] and, later, was shown to also secrete prolactin (Prl) [19]. This cell line has been suggested not to be a homogeneous population, but rather functionally heterogeneous based on the presence of a subset of both Gh-secreting and Prl-secreting cells by reverse hemolytic plaque assays and altered proportions of secreted Gh and Prl in response to different stimuli [20].

In this study, to clarify the endogenous AIP function, we generated an *Aip* knockout cell line from GH3 cells, termed GH3-FTY cells, using the CRISPR/Cas9 system [21]. We then characterized the capability of GH3-FTY cells for proliferation and Gh secretion *in vitro* and *in vivo* through comparisons with the parental line. We also investigated the underlying mechanism of increased Gh secretion and proliferation of GH3-FTY cells.

## Materials and Methods

### Cell line and sequence analysis of Aip

A rat pituitary tumor cell line, GH3, (ATCC, Manassas, VA) was cultured in F-12K medium (Life Technologies, Carlsbad, CA) containing 15% horse serum, 2.5% fetal bovine serum, 100 unit/ml penicillin, and 100 μg/ml streptomycin. The *Aip* sequence in GH3 cells was first confirmed. Genomic DNA was extracted using the Wizard genomic DNA purification kit (Promega, Madison, WI) and the exons containing splicing sites of adjacent introns were amplified by PCR using KOD FX (TOYOBO, Osaka, Japan) and directly sequenced using PCR primers detailed in **S1 Table**. The sequence was compared with that of *Aip* (NM_172327.2).

### Aip-knockout clone

*Aip*-mutant clones were created using the GeneArt CRISPR Nuclease Vector Kit (Life technologies) according to the manufacturer’s protocol. Because we identified a heterozygous frameshift mutation causing a premature stop codon that was causative of familial acromegaly (unpublished data), we targeted exon 4 using CRISPR/Cas9 in this study. c.490–492 and c.493–512 of *Aip* (NM_172327.2) were chosen as the protospacer adjacent motif and the target sequence, respectively. The top strand, 5′-TGCCCATGGGTCCTGCTGTTTT-3′ and the bottom strand, 5′-AGCAGGACCCATGGGCACGGTG-3′ were annealed and cloned into the CRISPR Nuclease Vector. The constructed CRISPR Nuclease Vector plasmid was nucleofected into GH3 cells using the Nucleofector Kit L (Lonza, Basel, Switzerland). Two days after nucleofection, CD4-positive cells were sorted using human CD4 MicroBeads (Milteni Biotec, Cologne, Germany). The sorted cells were seeded into 96-well plates at one cell per well and cultured. Genomic DNA was extracted from each clone and exon 4 of *Aip* was amplified and directly sequenced. Other exonic sequences of *Aip* including exon–intron boundaries of the target clones were also directly sequenced.

### Quantitative real-time PCR (qPCR) and the lentivirus vector

To examine mRNA expression levels, 2×10^5^ GH3 cells, Clone 1 (GH3-FTY), or Clone 2 were seeded into each well of 24-well plates and total RNA was prepared after a 48-h incubation using the RNeasy Kit (Qiagen, Venlo, the Netherlands). cDNA was prepared from 1 μg total RNA using the QuantiTect RT Kit (Qiagen), and qPCR was performed using Light Cycler 2.0 (Roche, Basel, Switzerland) and SYBR Premix Ex Taq II (Takara, Otsu, Japan). Primers are listed in **S1 Table**. PCR conditions are available on request.

Forced expression of *Aip* was performed by a lentivirus-mediated method as previously described [22]. Rat *Aip* cDNA synthesized and verified by Eurofins Genomics Co., Ltd (Tokyo, Japan) was used to construct a lentivirus vector plasmid (CSII-EF-IRES2-Venus) (RIKEN BRC) and prepared lentivirus. To investigate whether exogenous *Aip* may reverse the phenomenon observed in GH3-FTY cells, cells were infected with lentivirus vector containing rat *Aip* cDNA (LV-Aip) or *gfp* cDNA (LV-GFP) as a control at multiplicity of infection (MOI) 25.

### Western blotting and immunoprecipitation

Western blotting was performed as described previously [23]. Both cell lines were cultured for 48 h, and lysed in RIPA Buffer (Sigma-Aldrich, Tokyo, Japan). Total protein was subjected to sodium dodecyl sulfate polyacrylamide gel electrophoresis (SDS-PAGE), transferred to PVDF membranes (BIO-RAD, Tokyo, Japan), and incubated with antibodies described in **S2 Table**. Each protein was detected using ECL Prime (GE Healthcare, Little Chalfont, UK).

For immunoprecipitation, cells were lysed in lysis buffer containing 50 mM Tris-HCl (pH 8.0), 150 mM NaCl, 1% NP-40, 1% protease inhibitor cocktail (nacalai tesque, Kyoto, Japan) and 1% phosphatase inhibitor cocktail (nacalai tesque). Cell lysate containing 1 mg of total protein was pre-incubated with protein A or G magnetic beads (CST) at 4°C for 1 h, and the supernatant was collected. This was then incubated with anti-pStat3 antibody (CST), anti-Stat3 antibody (CST), anti-Aip antibody (Novus), normal rabbit IgG, or normal mouse IgG at 4°C overnight, and then protein A or G magnetic beads were added and incubated at 4°C for 1 h. The magnetic beads were washed five times with lysis buffer, and 2 × SDS-PAGE sample buffers was added to the magnetic beads, and incubated at 96°C for 5 min. The immunoprecipitated sample was then subjected to western blotting as described above.

### cAMP concentration

2×10^5^ of GH3 or GH3-FTY cells were plated in each well of 24-well plates, cultured for 24 h, and the intracellular cAMP concentration was determined using the Cyclic AMP Select EIA Kit (Cayman Chemical, Ann Arbor, MI) according to the manufacturer’s protocol.

### Cell proliferation

A total of 1×10^4^ of GH3 or GH3-FTY cells were plated in each well of 96-well plates, and cell numbers at 0, 24, and 48 h were evaluated using a Cell Counting Kit-8 (Dojindo, Kumamoto, Japan).

The BrdU assay was performed using the BrdU Cell Proliferation ELISA Kit (Abcam, Cambridge, UK). A total of 1×10^4^ cells were plated in each well of 96-well plates and cultured for 24 h, with BrdU incorporated for the final 2 h. The assay was performed according to the manufacturer’s protocol.

### Histological analysis

Hematoxylin-eosin (HE) staining and immunostaining were performed as described previously [23]. Microscopic observation was carried out using a Bio-REVO BZ-9000 fluorescence microscope (Keyence, Tokyo, Japan). Antibodies are described in **S2 Table**. Sections were counterstained with DAPI and visualized with an LSM710 inverted confocal microscope (Carl Zeiss, Tokyo, Japan).

### Gh measurement

A total of 2×10^5^ cells were plated into each well of 24-well plates and cultured in 1ml of F-12K medium containing 15% horse serum and 2.5% fetal bovine serum (FBS). Both horse serum and FBS were used after the treatment with dextran-coated charcoal (Sigma-Aldrich). After 24 h culture, the Gh concentration of the medium was measured by a Growth Hormone, Rat, EIA Kit (Bertin Pharma, Paris, France) and calibrated by RNA content. Total RNA was extracted and its content was determined by absorbance at 260 nm.

### Cell cycle analysis

Cell cycle profiles were analyzed by flow cytometry [24]. Cell nuclei were stained with a Cycletest plus DNA reagent kit (BD Biosciences, Tokyo, Japan), and propidium iodide fluorescence was acquired using FACSVerse (BD Biosciences).

### Analysis of GH3 or GH3-FTY xenografts

All animal protocols were approved by the Animal Care and Use Committee of Fukuoka University (approval number: 1501799). Male BALB/c-nu mice (Charles River Laboratories, Inc., Yokohama, Japan) were maintained on a 12-h light, 12-h dark cycle and given free access to water and a normal diet (Kyudou Co., Tosu, Japan). GH3 or GH3-FTY cells (5 × 10^5^) in 0.5 ml of F-12K medium were subcutaneously inoculated into the posterior flank region of 5-week-old mice (n = 8). Control mice were inoculated with 0.5 ml of F-12K medium only (n = 5). During the experimental procedure, mice were monitored and weighed twice a week. Four or eight weeks after inoculation, the mouse longitudinal length was measured using a Skyscan1178 CT scan for small animals (Bruker Corporation, Billerica, MA) under anesthesia which was achieved by an intraperitoneal injection of pentobarbital (30–40 mg/kg). Mice were sacrificed under isoflurane inhalation anesthesia at 8 weeks, and tumors, livers, and plasma were collected. The tumor volume was calculated as length × width squared × 0.52, as previously reported [25]. The plasma concentration of Gh was measured by the Growth Hormone, Rat, EIA Kit (Bertin Pharma). Igf-1 was measured by a Quantikine ELISA mouse/rat IGF-1 kit (R&D systems, Minneapolis, MN).

To evaluate glucose metabolism, glucose tolerance testing (GTT) and insulin tolerance testing (ITT) were performed at 6 weeks. In the GTT, after 15 h fasting, 2 g/kg body weight (BW) glucose was administered by intraperitoneal injection, and blood glucose (BG) at 0, 15, 30, 60, and 120 min was measured by a Glutest Mint (Sanwa Chemistry Co., Ltd., Hiratsuka, Japan). Plasma insulin levels at 0, 15, 30, and 60 min were measured using a hypersensitive mouse insulin kit (Morinaga Institute, Inc., Yokohama, Japan). ITT was performed 7 weeks after inoculation by the intraperitoneal administration of 0.75 U/kg BW Novolin R (Novo Nordisk Pharma Ltd., Tokyo, Japan) after 3-h fasting; BG was then measured at 0, 15, 30, and 60 min.

We then repeated these experiments (n = 3 in each group) using an endpoint of 4 weeks. Tumors were therefore obtained at 4 weeks and subjected to histological analysis.

### Statistical analysis

All experiments were performed at least three times; all display items are representative of the experiments. Statistical analysis was carried out using the unpaired two-tailed *t*-test and one-way analysis of variance (ANOVA) with Tukey’s post-hoc test as appropriate using GraphPad Prism software. All data are expressed as means ± standard error of the mean (SEM). *P*<0.05 was considered to be statistically significant.

## Results

### Intact Aip was disrupted in GH3-FTY cells

**Fig 1:**
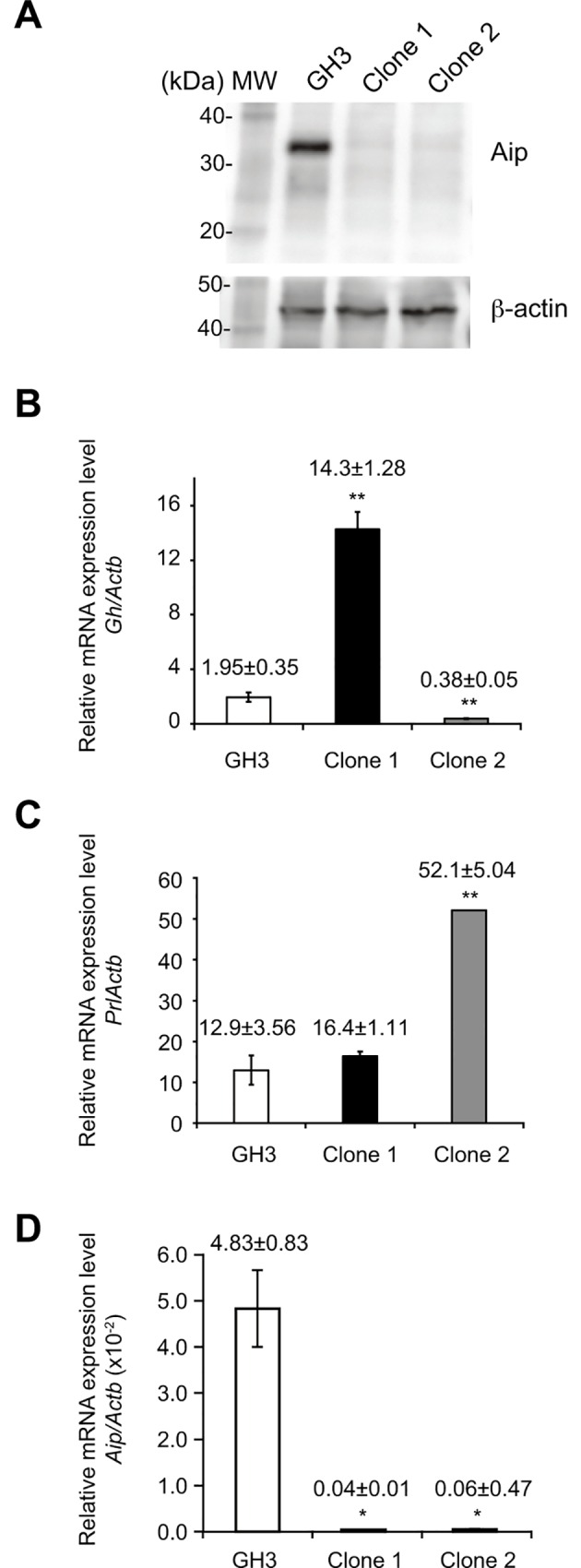
Screening of *Aip*-disrupted GH3 clones generated by CRISPR/Cas9. The detailed mutations of clones 1 and 2 are described in the Results section and **S1 Fig**. Clone 1 corresponds to GH3-FTY cells intensively investigated in the present study. (**A**) Western blot analysis of Aip protein in GH3 and mutant GH3 clones (clones 1 and 2). Fifteen μg of protein from cell lysates was subjected to SDS-PAGE and immunoblotted with antibodies against Aip and beta-actin. Each target protein was visualized using VersaDoc 5000 (BIO-RAD, Tokyo, Japan). (**B, C**) The relative expression of *Gh* mRNA and *Prl* mRNA to *Actb* mRNA is shown, respectively. Data were compared using the unpaired two-tailed *t*-test. **P<0.01 (clone 1 or 2) vs GH3. (**D)** The relative expression of *Aip* mRNA to *Actb mRNA* in cultured clone 1 (GH3-FTY cells) and clone 2 is shown. Data were compared using the unpaired two-tailed *t*-test. * *P*<0.05 (clone 1 or 2) vs GH3.

Exonic sequencing of *Aip* exon–intron boundaries in GH3 cells revealed complete homology with rat *Aip* cDNA (NM_172327.2) and no mutations were found. Screening of *Aip*-mutant after genome editing identified two clones that contained compound heterozygous mutations of both *Aip* alleles, which resulted in truncated Aip proteins. While the estimated molecular weight of 37 kDa was detected in GH3 cells containing wild-type *Aip*, no corresponding band was detected in clones 1 and 2 (**Fig 1A**). Despite the genetic disruption of *Aip* and the complete disappearance of Aip protein, the two clones did not show the same phenotype with respect to Gh and Prl synthesis, as determined by qPCR (**Fig 1B and 1C**). This is probably because of the heterogeneous population of GH3 cells, as reported in previous studies [20, 26].

Of the two *Aip*-disrupted GH3 clones, only one (clone 1), termed GH3-FTY, showed a 7.3-fold increase in Gh synthesis but only a 1.3-fold increase in Prl synthesis compared with GH3 cells, indicating that clone 1 corresponds to a somatotroph cell line. However, clone 2 showed relatively higher levels (4.0-fold) of Prl synthesis than GH3 cells, while Gh synthesis was only 0.2-fold that of GH3 cells, indicating that this clone was more representative of a lactotroph cell line. Interestingly, relative mRNA expression levels of mutant *Aip* in GH3-FTY cells (clone 1) and clone 2 were extremely low compared with that of GH3 cells (*P* = 0.0098 and *P* = 0.010, respectively) (**Fig 1D**), suggesting that the stability of *Aip* mRNAs derived from these two clones is also decreased. In subsequent studies, we only focused on the characterization of clone 1 (GH3-FTY). Characterization of clone 2 will be reported in the future.

The *Aip* sequence and the amino acid sequence of clone 1 (GH3-FTY) are shown in **S1A Fig**. GH3-FTY contained compound heterozygous frameshift mutations in the form of an adenine insertion at c.496 and adenine and guanine deletions at c.496-497, causing premature stop codons at codons 173 and 172, respectively (**S1A Fig**). The predicted truncated Aip proteins completely lacked TPR domains (**S1B Fig**). Clone 2 contained compound heterozygous mutations of an adenine insertion at c.496 (as observed in clone 1) in one allele and a deletion of guanine at c.497 in the other allele (data not shown).

### GH3-FTY cells showed dramatic Gh secretion levels compared with GH3 cells

**Fig 2:**
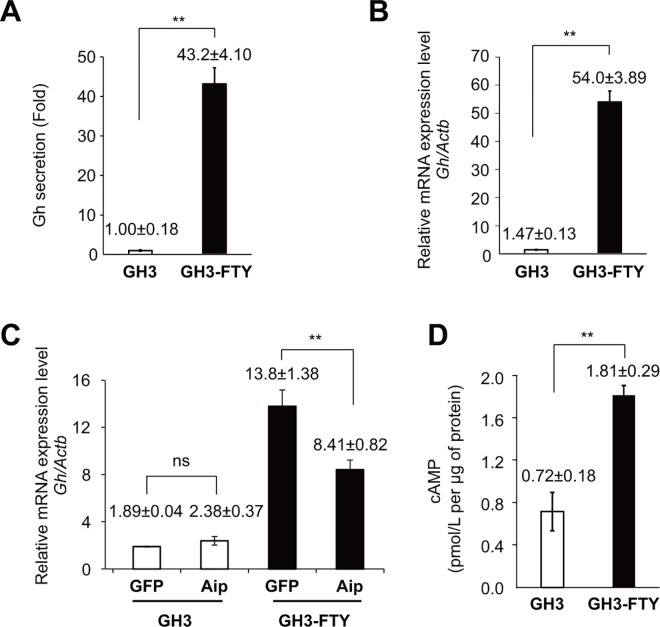
Gh secretion in the cultured medium, *Gh* mRNA levels before and after exogenous *Aip* expression, and intracellular cAMP content in cultured GH3 and GH3-FTY cells. (**A**) Gh secretion in the media from cultured GH3 and GH3-FTY cells was measured and expressed as a fold increase. (**B**) *Gh* mRNA levels in cultured GH3 and GH3-GTY cells were calculated relative to *Actb* (internal control) using the ∆Ct method. (**C**) The effect of lentivirus (LV)-mediated forced expression of exogenous *gfp* as a control or *Aip* on *Gh* mRNA levels. GFP and Aip indicate LV-GFP and LV-Aip, respectively. (**D**) The intracellular cAMP content of both cultured cells was measured as described in the Methods. Data were compared using the unpaired two-tailed *t*-test. **P<0.01. ns, not significant.

GH3-FTY cells showed 20–43-fold stronger Gh secretion levels than GH3 cells. **Fig 2A** shows a typical example (*P* = 0.0024). *Gh* mRNA levels in GH3-FTY cells were 7.3–36.7-fold higher than in GH3. **Fig 2B** shows a typical example (*P* = 0.0018). The increase in *Gh* mRNA expression of GH3-FTY cells was significantly suppressed by lentivirus (LV)-mediated exogenous Aip expression (*P* = 0.0082), while the level of G*h* mRNA in GH3 cells was unchanged by the same treatment, suggesting that the endogenous Aip function is specifically disrupted in GH3-FTY cells (**Fig 2C**). cAMP levels in GH3-FTY cells were significantly higher than in GH3 cells (**Fig 2D**).

### GH3-FTY cells showed increased proliferation in culture

**Fig 3:**
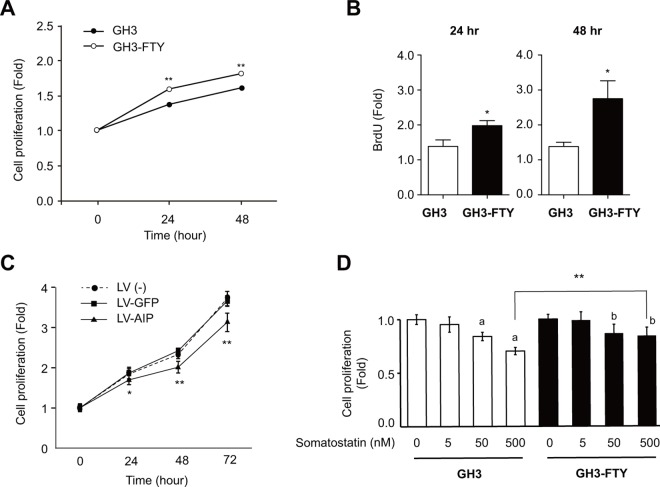
Proliferation of GH3-FTY versus GH3 cells. (**A**) The numbers of cultured GH3 and GH3-FTY cells at 24 h and 48 h were compared by the cell counting kit. The value at each time point was calibrated by the cell numbers at the start of the experiment (0 h). Data were compared using the unpaired two-tailed *t*-test. *P<0.05. ns, not significant. (**B**) BrdU assay at 24 h and 48 h. Data were compared using the unpaired two-tailed *t*-test. *P<0.05. (**C**) Effect of lentivirus (LV)-mediated forced expression of exogenous *Aip* (LV-AIP) or *gfp* (LV-GFP) as a control on the proliferation of cultured GH3-FTY cells. LV (-) indicates GH3-FTY cells uninfected with lentivirus. Statistical significance was observed only between LV-GFP and LV-Aip (*P<0.05; **P<0.01), but not between LV (-) and LV-GFP. ns, not significant. (**D**) Sensitivity to somatostatin (0–500 nM) was compared between cultured GH3 and GH3-FTY cells. Data were compared using the unpaired two-tailed *t*-test. **P<0.01, 500 nM somatostatin-treated GH3-FTY vs 500 nM somatostatin-treated GH3. ^a^ *P*<0.01 vs 0 nM somatostatin-treated GH3. ^b^*P*<0.01 vs 0 nM somatostatin-treated GH3-FTY.

The proliferation of cultured GH3 and GH-FTY cells as determined by a cell counting kit is shown in **Fig 3A**. The proliferation rate of GH3-FTY cells was not exceptional but was significantly increased compared with parental GH3 cells after 24 and 48 h (*P* = 0.00036 and *P* = 0.00066, respectively). This was also confirmed by a BrdU assay at 24 and 48 h (*P* = 0.0294 and *P* = 0.0271, respectively) (**Fig 3B**). Cell cycle analysis revealed that GH3-FTY cells showed an increased S phase relative to GH3 cells in the presence or absence of serum stimulation, although was slightly more evident in the absence of serum (**Table 1**). The sub-G1 fraction, namely the apoptotic cell fraction, was not observed under any conditions in GH3 and GH3-FTY cells. Importantly, the increased cell proliferation of GH3-FTY was significantly reversed by LV-mediated exogenous Aip expression, while it was unchanged by LV-mediated GFP expression (**Fig 3C**). Somatostatin dose-dependently suppressed the proliferation of GH3 and GH3-FTY cells. However, GH3-FTY cells were less sensitive to somatostatin than GH3 cells (**Fig 3D**).

**Table 1:** Cell cycle analyses of GH3 and GH3-FTY in the presence or absence of serum.

cell	serum	G0/G1 (%)	S (%)	G2/M (%)
GH3	+	59.0	29.0	9.58
GH3	-	84.6	17.7	13.8
GH3-FTY	+	47.0	34.7	10.5
GH3-FTY	-	58.0	25.9	12.2

Cells were pre-cultured in medium without serum for 24 h and then either cultured in medium containing serum for another 24 h or cultured in serum-free medium for 48 h. Propidium iodide fluorescence levels of 2×10^4^ events were acquired using a flow cytometer. G0/G1, S, and G2/M fractions were calculated from the resulting cell cycle profile using FlowJo (TOMY Digital Biology Co., Ltd., Tokyo, Japan).

### GH3-FTY cells showed increased phosphorylation of Stat3 (Tyr705)

**Fig 4:**
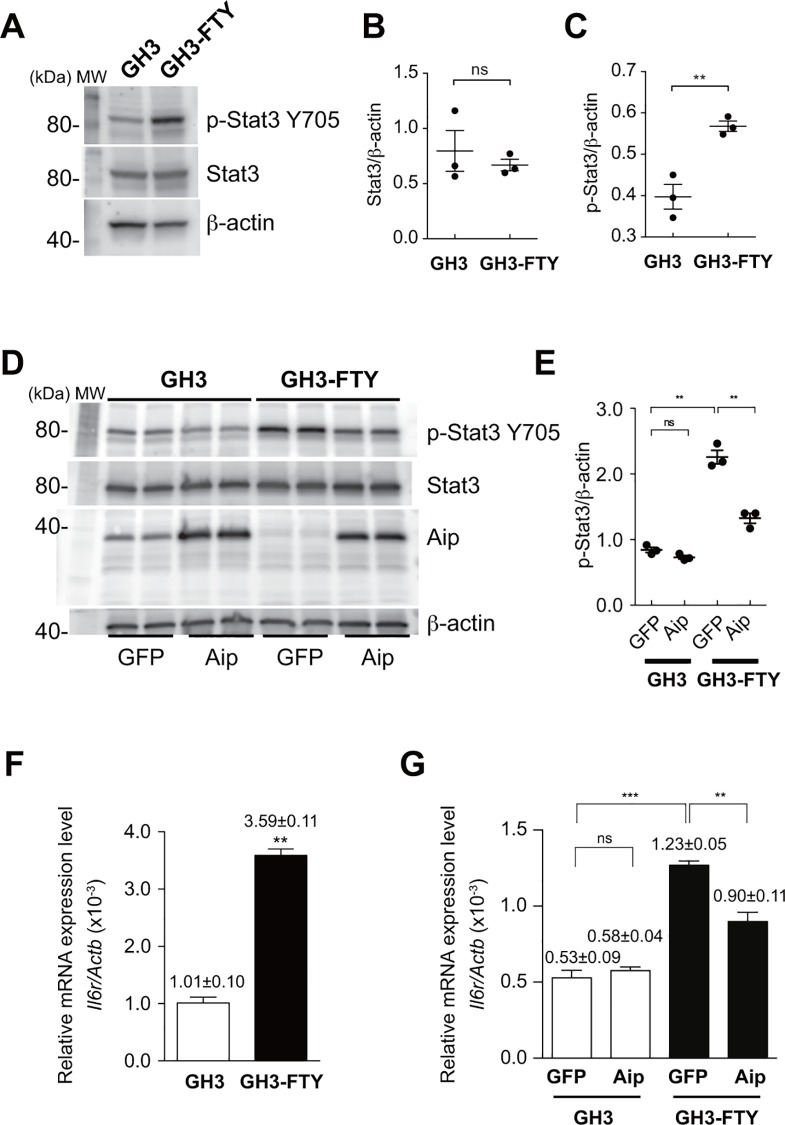
Western blot analysis of p-Stat3 (Tyr705) and Stat3 in cultured GH3 and GH3-FTY cells. (**A**) Western blot analysis of p-Stat3 (Tyr705) and Stat3. Twenty μg protein from cell lysates was separated by SDS-PAGE and immunoblotted with antibodies against p-Stat3, Stat3, and beta-actin. (**B**) Statistical evaluation of Stat3/ beta-actin expression between cultured GH3 and GH3-FTY cells. Data were compared using the unpaired two-tailed *t*-test. ns, not significant. The intensity of each detected band was analyzed using the image analysis software Quantity One (BIO-RAD), and the Stat3/ beta-actin ratio was calculated. (**C**) Statistical evaluation of p-Stat3/ beta-actin expression between cultured GH3 and GH3-FTY cells. Data were compared using the unpaired two-tailed *t*-test. **P<0.01. (**D)** Western blot analysis of p-Stat3 and Stat3. The effect of lentivirus (LV)-mediated forced expression of exogenous *Aip* (LV-AIP) or *gfp* (LV-GFP) as a control on p-Stat3 expression was examined. Twenty μg protein from cell lysates was subjected to SDS-PAGE and immunoblotted with antibodies against p-Stat3 and beta-actin. (**E)** Statistical evaluation of p-Stat3. GFP and Aip indicate LV-GFP and LV-Aip, respectively. Data were compared using the unpaired two-tailed *t*-test. **P<0.01. ns, not significant. (**F**) *Il6r* mRNA levels relative to *Actb* mRNA levels as determined by qPCR. (**G**) *Il6r* mRNA levels relative to *Actb* mRNA levels as determined by qPCR before and after lentivirus (LV)-mediated exogenous expression of *Aip* (LV-Aip) or *gfp* (LV-GFP) as a control. GFP and Aip indicate LV-GFP and LV-Aip, respectively. Data were compared using the unpaired two-tailed *t*-test. *P<0.05, **P<0.01. ns, not significant.

With regards to the mechanism of increased Gh secretion, we investigated the involvement of Stat3 using Western blot analysis. As shown in **Fig 4A** and **4B**, the expression of Stat3 was unchanged between GH3 and GH3-FTY cells. However, p-Stat3 was significantly increased in GH3-FTY compared with GH3 **cells** (*P* = 0.0062) (**Fig 4A** and **4C**). Importantly, the upregulation of p-Stat3 in GH3-FTY cells was reversed by LV-mediated forced expression of *Aip* (*P* = 0.0019) (**Fig 4D** and **4E**). To investigate whether Aip and p-Stat3 or Stat3 form protein complex, we performed immunoprecipitation assay. Neither of the cell lysates from GH3 or GH3-FTY cells immunoprecipitated with an anti-Aip antibody could be coprecipitated with anti-p-Stat3 or anti-Stat3 antibodies. Additionally, neither of the cell lysates from GH3 or GH3-FTY cells immunoprecipitated with anti-p-Stat3 or Stat3 antibodies could be coprecipitated with an anti-Aip antibody. These results suggest that Aip does not form a complex with p-Stat3 or Stat3 (data not shown). Next, the mRNA expression of interleukin 6 receptor (*Il6r*), an upstream receptor of Stat3, was examined by qPCR. Interestingly, *Il6r* mRNA was found to be upregulated in GH3-FTY cells compared with GH3 cells (*P*<0.0001) (**Fig 4F**), and this increase was significantly reversed by the LV-mediated forced expression of *Aip* (*P* = 0.0053) (**Fig 4G**).

### GH3-FTY cells showed less sensitivity to somatostatin in association with decreased expression of somatostatin receptor 2 (Sstr2) and Zac1

**Fig 5:**
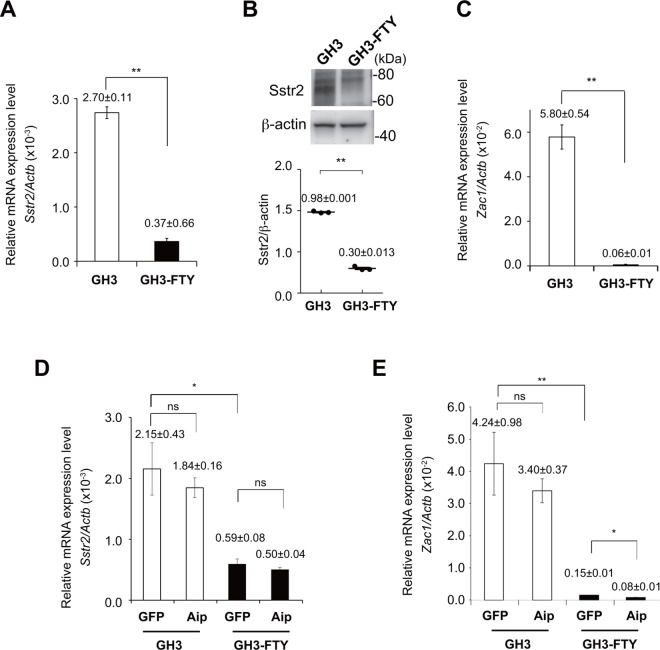
Comparison of *Sstr2*, and *Zac1* mRNA expression and Sstr2 expression levels by western blot in cultured GH3 and GH3-FTY cells. (**A**) *Sstr2* mRNA levels relative to *Actb* mRNA levels as determined by qPCR. (**B**) Western blot analysis of Sstr2 and beta-actin in cultured GH3 and GH3-FTY cells. Twenty μg protein from cell lysates was separated by SDS-PAGE and immunoblotted with antibodies against Sstr2 and beta-actin (upper panel). Statistical evaluation of Sstr2/ beta-actin expression between cultured GH3 cells and GH3-FTY cells (lower panel). (**C**) *Zac1* mRNA levels relative to *Actb* mRNA levels as determined by qPCR. (**D**) *Sstr2* mRNA levels relative to *Actb* mRNA levels as determined by qPCR after lentivirus (LV)-mediated exogenous expression of *Aip* (LV-Aip) or *gfp* (LV-GFP) as a control. GFP and Aip indicate LV-GFP and LV-Aip, respectively. (**E**) *Zac1* mRNA levels relative to *Actb* mRNA levels as determined by qPCR after lentivirus (LV)-mediated exogenous expression of *Aip* (LV-Aip) or *gfp* (LV-GFP) as a control. GFP and Aip indicate LV-GFP and LV-Aip, respectively. Data were compared using the unpaired two-tailed *t*-test. *P<0.05, **P<0.01. ns, not significant.

As shown in **Fig 3D**, GH3-FTY cells were less sensitive to somatostatin as a suppressor of cell proliferation than GH3 cells. This was supported by findings that Sstr2 expression levels were dramatically decreased in GH3-FTY cells compared with GH3 cells at both mRNA and protein levels (*P*<0.0001 and P<0.0001, respectively) (**Fig 5A** and **5B**). In relation to Aip function, the mRNA expression levels of *Zac1*, a possible inhibitory factor of GH secretion and cell proliferation [27], was significantly decreased in GH3-FTY cells compared with GH3 cells (*P* = 0.0015) (**Fig 5C**). However, the decrease in mRNA levels of *Sstr2* and *Zac1* was not reversed by LV-mediated exogenous expression of *Aip* (**Fig 5D** and **5E**).

### GH3-FTY xenografts formed more anaplastic tumors than GH3 xenografts

**Fig 6:**
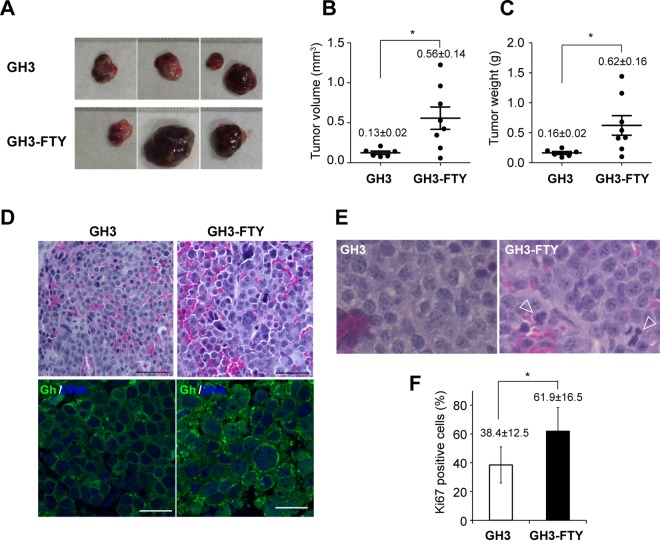
Analysis of tumors in GH3 or GH3-FTY-xenografted mice. (**A**) Actual appearance of tumors removed from control mice, GH3 mice, and GH3-FTY mice. (**B**) Tumor volume and (**C**) tumor weight 8 weeks after inoculation. Data were compared using the two-tailed unpaired *t*-test. *P<0.05. (**D**) Typical tissue histology of somatotroph cell tumors in GH3 or GH3-FTY mice by HE staining (upper two panels) and immunohistochemical analysis (lower two panels) at 4 weeks. Bar, 50 μm. Staining of Gh (green) and nucleus (DAPI, blue) was observed using confocal microscopy. A larger N/C ratio and anisonucleosis in GH3-FTY cells relative to GH3 cells were observed. More prominent Gh vesicles were observed in GH3-FTY compared with GH3 mice. Bar, 20 μm. (**E**) Typical tissue histology of tumors in GH3 and GH3-FTY mice by HE staining at 8 weeks. Arrowheads indicate abnormal mitotic cells. (**F**) Ki67 scores of both tumors were statistically compared. Data were compared using the two-tailed unpaired *t*-test. *P<0.05.

Xenografts of GH3-FTY cells and GH3 cells formed tumors at the position of cell inoculation in nude mice (**Fig 6A**). Eight weeks after inoculation, the average tumor volume of GH3-FTY xenografts (GH3-FTY mice) was 4.31-fold larger than that of GH3 xenografts (GH3 mice) (*P* = 0.0215) (**Fig 6B**). The average tumor weight of GH3-FTY mice was 3.88-fold larger than that of GH3 mice (*P* = 0.0331) (**Fig 6C**). HE staining at 4 weeks revealed that tumors in GH3-FTY mice showed more mitotic features than those in GH3 mice in that GH3-FTY mouse tumors were comprised of cells showing a relatively larger nuclear/cytoplasm (N/C) ratio with anisonucleosis (**upper panels in Fig 6D**). Immunostaining of Gh and DAPI staining of tumor nuclei from GH3-FTY mice at 4 weeks supported these findings. Additionally, Gh was predominantly stained in secretory vesicles, and this staining was more apparent in GH3-FTY cells compared with GH3 cells (**lower panels in Fig 6D**). Mitotic features in tumors from GH3-FTY mice at 8 weeks after inoculation were less obvious because of the increased level of necrosis (data not shown). Abnormal mitosis was frequently observed in the tumors of GH3-FTY mice compared with those of GH3 mice (**Fig 6E**). In accordance with these findings, GH3-FTY mouse tumors showed significantly increased Ki67 scores compared with those in GH3 mice (*P* = 0.0084) (**Fig 6F**).

### GH3-FTY mice show a larger size and higher Gh secretion compared with GH3 mice

**Fig 7:**
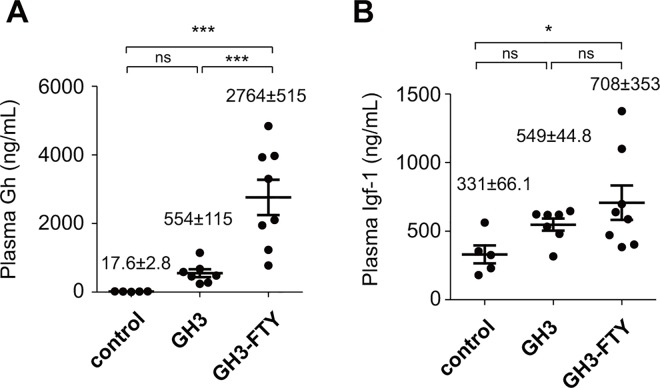
Plasma Gh and Igf-1 levels in GH3 and GH3-FTY mice. BALB/c-nu mice were inoculated with GH3 or GH3-FTY cells. Control mice were inoculated by the medium only. Plasma levels of Gh (**A**) and Igf-1 (**B**) at 8 weeks after inoculation. Data were compared using the one-way ANOVA with Tukey’s post-hoc test. *P<0.05, ***P<0.001. ns, not significant.

**Fig 8:**
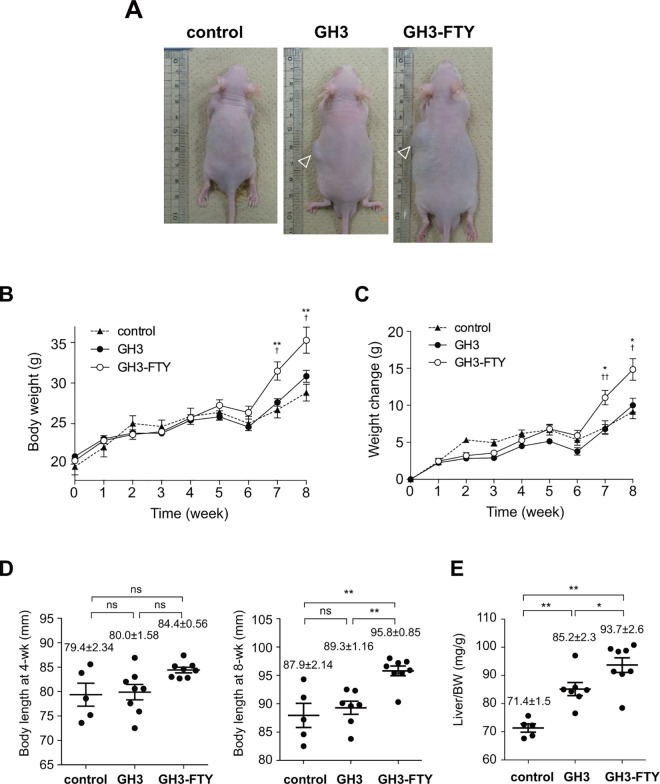
Body weight, weight change, body length, and liver weight in GH3 and GH3-FTY mice. (**A**) Actual appearance of control, GH3, and GH3-FTY mice. Arrowheads indicate xenograft tumor. (**B**) Time course of body weight changes. A significant difference between GH3-FTY and control mice was observed from 7 weeks after inoculation. No significant difference was observed between GH3 and control mice during the observation period. Statistical comparison of changes in body weight (**C**), body length (**D**), and liver weight (**E**) in control, GH3, and GH3-FTY mice. Data were compared using the one-way ANOVA with Tukey’s post-hoc test. *P<0.05, **P<0.01 vs control. †*P*<0.05, ††*P*<0.01 vs GH3. ns, not significant.

Eight weeks after inoculation, several phenotypic differences between GH3 and GH3-FTY mice were compared. Plasma Gh levels were 554±115 ng/mL (n = 7) and 2764±515 ng/mL (n = 8), respectively, which were both higher than Gh levels in control mice (17.6±2.8 ng/mL) (n = 5). The plasma Gh level in GH3-FTY mice was significantly higher than that of GH3 mice (*P*<0.0001) (**Fig 7A**). Plasma Igf-1 levels at 8 weeks were 549±44.8 ng/mL (n = 7) and 708±353 ng/mL (n = 8), respectively (**Fig 7B**). Although plasma Igf-1 levels of GH3-FTY mice were significantly higher than those of control mice (*P*<0.05), they were not significantly different between GH3 and GH3-FTY mice. GH3-FTY mice were larger than GH3 and control mice (**Fig 8A**). Body weights are shown in **Fig 8B**; average weights of control, GH3, and GH3-FTY mice at 8 weeks after inoculation were 28.8±2.3 g (n = 5), 30.8±3.0 g (n = 7), and 35.3±5.3 g (n = 8), respectively (*P*<0.05, GH3-FTY vs. control or GH3). No significant difference was observed between control and GH3 mice, while GH3-FTY mice were significantly heavier from 7 weeks after inoculation than both control and GH3 mice. Weight changes of GH3-FTY mice were significantly increased compared with control and GH3 mice after 7 week from inoculation, and the average weight changes of control, GH3, and GH3-FTY mice at 8 weeks after inoculation were 9.12.8±0.93 g (n = 5), 10.0±0.94 g (n = 7), and 14.8±1.45 g (n = 8), respectively (*P*<0.05, GH3-FTY vs. control or GH3) (**Fig 8C**). Longitudinal lengths at 8 weeks after the xenograft injection in control, GH3, and GH3-FTY mice were 87.9±2.1 mm (n = 5), 89.3±1.2 mm (n = 7), and 95.8±0.9 mm, (n = 8) respectively (*P*<0.01, GH3-FTY vs. control or GH3) (**Fig 8D**). No significant difference between control and GH3 mice was observed (**Fig 8D**). Liver weights (mg/gBW) of GH3-FTY at 8 weeks (n = 8) were also increased compared with those of control (n = 5) (*P*<0.0001) and GH3 mice (n = 7) (*P*<0.05) (**Fig 8E**).

### GH3-FTY mice showed stronger insulin resistance compared with GH3 mice

**Fig 9:**
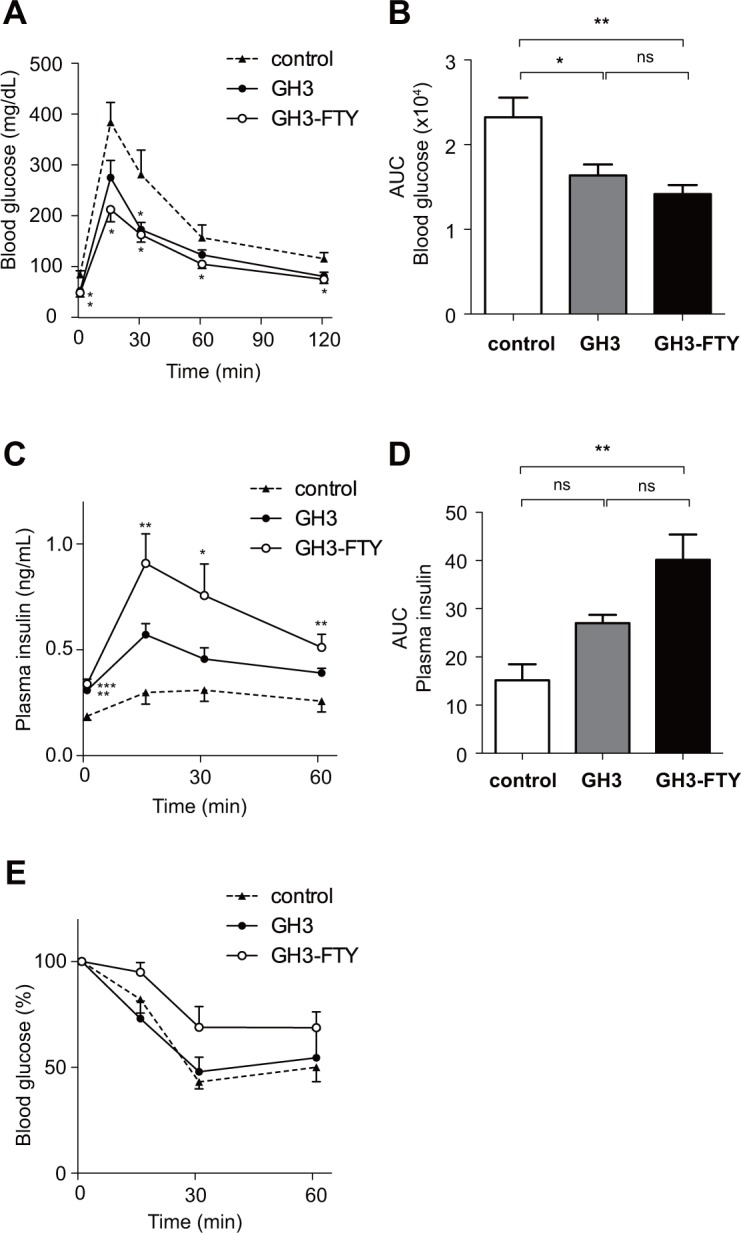
Glucose metabolism of control, GH3, and GH3-FTY mice after xenograft inoculation. Six weeks after xenograft inoculation, GTT was performed. Changes in BG levels (**A**), AUC of blood glucose (**B**), plasma insulin levels (**C**), and AUC of plasma insulin levels (**D**). (**E)** Seven weeks after inoculation, ITT was performed. Data were compared using the one-way ANOVA with Tukey’s post-hoc test. *P<0.05, **P<0.01 between GH3 or GH3-FTY and control mice. ns, no significant change.

To compare the biological effect of GH3 and GH3-FTY on glucose metabolism, GTT and ITT were performed (**Fig 9)**. In GTTs performed 6 weeks after the xenograft, BG levels in GH3 and GH3-FTY mice at 0, and 30 min were significantly decreased compared with those of control mice (*P*<0.05). No significant differences in BG levels between GH3 and GH3-FTY mice were evident at any time point and there was no significant difference in the area under the curve (AUC) (**Fig 9A** and **9B**). Insulin levels at 0 min were significantly increased in GH3 and GH3-FTY mice compared with controls (*P*<0.01). Furthermore, insulin levels in GH3-FTY mice at 15, 30 and 60 min, as well as the AUC, were significantly increased compared with control mice (*P*<0.05) (**Fig 9C** and **9D**). However, insulin levels at 15, 30 and 60 min, as well as the AUC between GH3 and GH3-FTY mice were not significantly different. GH3-FTY mice showed higher tendency of glucose levels than control or GH3 mice in an ITT performed 7 weeks after inoculation. However, the difference was not statistically significant (*P* = 0.00694, 0.0802 and 0.4038 at 15, 30 and 60 min, respectively) (**Fig 9E**).

## Discussion

In this study, we used the CRISPR/Cas9 system to generate a new cell line from GH3 cells, which lacked functional *Aip* and which we named GH3-FTY cells. This cell line showed dramatically increased (20–43-fold) Gh secretion compared with GH3 cells. The fluctuating and unstable secretion of basal Gh and varied Gh/Prl secretion ratio from cultured GH3 cells, even from the same ATCC lot, has been previously reported [26]. This is thought to be partly because GH3 cells are not a homogeneous cell clone [20, 26]. Even though such an unstable nature of GH3 cells was reflected to some extent in our results, the tremendous capability of Gh secretion from GH3-FTY cells over GH3 cells far exceeds the previously reported variable levels in GH3 cells (around 2–6 times) [26] and caused more potent stable biological activity in the xenograft model. Because GH3-FTY cells were cloned from a single GH3 cell after genome editing, they appear to be a real homogeneous cell clone.

The proliferative capability of GH3-FTY cells did not differ greatly from that of parental GH3 cells, but the increased DNA synthesis of GH3-FTY relative to GH3 cells was supported by the BrdU assay and the finding of an increased S phase relative to G1 phase in cell cycle analysis. The enhanced Gh production from cultured GH3-FTY cells relative to GH3 cells might promote the increase in S phase even in the absence of serum. Importantly, forced expression of *Aip* using the lentivirus system in cultured GH3-FTY cells partially reversed the increased *Gh* mRNA expression and almost completely reversed the changes in cell proliferation and the cell cycle profile. While the exact reason for partial reversibility in increased Gh synthesis remains unclear, it may reflect the insufficient expression levels of exogenous *Aip* or the heterogeneous or fluctuating capability of Gh synthesis of the parental GH3 cell line. Nevertheless, our results indicate that complete loss of Aip function in Gh-producing cells leads to increased Gh secretion and cell proliferation. Homozygous *Aip*-knockout in mice is lethal, thus making it difficult to confirm endogenous *Aip* function *in vivo*, although *Aip*-knockout mouse embryonic fibroblasts (MEFs) are available [13]. However, our cell line is the first demonstration of an *Aip*-knockout pituitary cell line. The increased cAMP production in GH3-FTY compared with GH3 cells appears to be involved in the observed Gh secretion, as evidenced by a previous report showing increased cAMP production in *Aip*-knockout MEFs or *Aip*-silenced GH3 cells [13].

The type I cytokine receptor family including the IL-6 receptor activates Janus kinase 2 upon ligand binding, which in turn activates Stat3 by phosphorylating Tyr705. Activated Stat3 (p-Stat3) undergoes homodimerization and nuclear translocation, and eventually leads to transcriptional regulation of target genes to modulate cell proliferation, survival, and differentiation [28]. In a recent report, the upregulation of Stat3 in pituitary somatotroph adenomas was reported to be associated with GH hypersecretion [29]. In this study, GH induced Stat3 phosphorylation in GH3 cells, suggesting an autoregulatory positive-feedback loop between Stat3 and GH in somatotroph tumor cells [29]. However, the involvement of Aip in this phenomenon has not been clarified. Interestingly, we revealed that despite the stable level of Stat3, p-Stat3 in GH3-FTY cells lacking *Aip* was significantly increased compared with GH3 cells. Additionally, the forced expression of *Aip* partially but clearly reversed the phenomenon. A complex formation of Aip and p-Stat 3 was not observed by immunoprecipitation assay, suggesting an indirect functional association between these proteins. Anyway these results suggest that the activation of Stat3 in GH3 cells is, at least partially, mediated by Aip. Thus, the autoregulatory positive loop between Stat3 and Gh may be more potentiated in GH3-FTY than in GH3 cells, resulting in the enormous production of Gh. Moreover, because *Il6r* mRNA expression was also increased in GH3-FTY cells, the above positive loop might be further augmented in some inflammatory situations *in vivo*.

GH3-FTY cells were less sensitive to somatostatin than GH3 cells. This may be partly explained by the reduced expression of Sstr2 in GH3-FTY cells. The antiproliferative effect of the somatostatin analog octreotide was suggested to be associated with the upregulation of ZAC1 expression [15], which was shown to correlate with the IGF-I response and tumor shrinkage [27]. Pituitary tumors with reduced AIP expression are reported to be frequently resistant to first-generation somatostatin analogs [9, 14]. However, it is not clear whether the reduced expression of AIP is associated with functional AIP inactivation. Furthermore, it is also not clear whether AIP inactivation is associated with decreased SSTR2 expression. In the present study, GH3-FTY cells showed reduced expression of Sstr2 as well as *Zac1* compared with GH3 cells, suggesting an association with *Aip* inactivation, as previously reported [9, 14, 15]. However, contrary to our prediction, the reduced expression of these mRNAs was not reversed by the exogenous expression of Aip. Thus, at least in our study, the direct association between Aip and Sstr2 or Zac1 seems to be unlikely. One alternative explanation is that levels of Sstr2 and Zac1 might be regulated by upstream molecules of Aip. We also cannot exclude the possibility that the low expression levels of Sstr2 and Zac1 are original characteristics of GH3-FTY cells which are unrelated to *Aip* inactivation. An inconsistent association between SSTR2 and AIP was clinically suggested from the finding that SSTR2 staining was not always reduced in pituitary somatotroph adenomas regardless of the presence or absence of *Aip* mutations [14]. We should also keep in mind that the antiproliferative effects of somatostatin can occur via Sstr2 through other pathways such as PI3k/Akt signaling [15], the MAPK cascade, activating tyrosine/serine phosphatases, and inhibiting adenylate cyclase activity [30–32] or even indirectly through reducing the release of growth-promoting factors such as IGF-I [33].

The biological function of the GH3-FTY cell line was determined *in vivo* by xenograft transplantation to nude mice. GH promotes body growth including that of the liver via STAT5b-mediated signaling [33]. Despite the increase in Gh secretion in GH-FTY compared with GH3 mice (~5-fold), body weight was significantly increased from 46 days after inoculation compared with control or GH3 mice. GH3 mice showed no such increase in body weight compared with controls during the observation period. The higher values of body length and liver weight in GH3-FTY mice compared with GH3 mice were confirmed at 8 weeks. It is likely that it takes time for various organs to become enlarged when we consider the observation of only a slight increase in the proliferation of GH3-FTY compared with GH3 cells.

The stronger biological functions of GH3-FTY mice over control mice were also confirmed in the evaluation of glucose metabolism. However, the difference in glucose metabolism was statistically not evident between GH3-FTY mice and GH3 mice. While the glucose tolerance in GTT performed at 6 weeks was almost equivalent between the two mice models, hyperinsulinemia in GTT or insulin resistance in ITT of GH3-FTY mice tended to be stronger than those of GH3. These results may reflect a time-dependent change in glucose tolerance associated with insulin resistance caused by Gh oversecretion. The simultaneous hypersecretion of Igf-1 may also modify the glucose tolerance by improving insulin sensitivity. These two mice models will be very useful in investigating the developmental process from insulin resistance to diabetes by GH oversecretion [34, 35].

The average tumor size of GH3-FTY mice was larger than that of GH3 mice, which may reflect the increased proliferative capability of GH3-FTY cells. Another possibility is the autocrine stimulation of the tumor by Gh, resulting in increased Igf-1 secretion in the circulation. Importantly, xenografted GH3-FTY somatotroph tumors showed more mitotic features than xenografted GH3 tumors, including a relatively larger N/C ratio, anisonucleosis, a more frequent occurrence of abnormal mitosis, and an increased Ki67 score. Such changes may be related to the continuous oversecretion of Gh on tumorigenesis, because Gh–IGF-1 promotion of cell proliferation is predominantly mediated by the MAPK pathway and stimulation of the antiapoptotic pathway [36]. Moreover, the disruption of *Aip* itself is reported to be associated with the oncogenic characteristic of GH3-FTY cells [3, 10–13]. Indeed, AIP expression was documented in invasive somatotropinoma tumors compared with noninvasive tumors [37].

In conclusion, we established the first known somatotroph cell line, GH3-FTY, in which endogenous *Aip* was completely disrupted using the CRISPR/Cas9 system. *Aip* inactivation promoted the overproduction of Gh in this cell line, probably through increased Stat3 activation compared with parental GH3 cells. GH3-FTY cells may be a useful model to demonstrate how Aip inactivation promotes somatotroph tumorigenesis and autonomous GH secretion. This cell line may also be useful for the screening of innovative drugs for acromegaly.

## Supporting Information

S1 Fig Nucleotide sequence of *Aip* exon 4 and schematic structure of Aip protein. (**A**) Nucleotide sequence of normal (wild-type) and mutant *Aip* exon 4 in GH3-FTY cells. GH3-FTY cells contained heterozygous mutants of an adenine insertion at c.496 and a two base deletion at c.496-497, causing premature stop codons at codon 173 (TGA) and codon 172 (TGA), respectively, in both *Aip* alleles. (**B**) Schematic of wild-type and mutant Aip structures found in GH3 cells.
(PDF)

S1 Table Primer list. Primers 1–6 were used to amplify *Aip*. Primers 7–13 were used for qPCR to determine the mRNA expression levels of respective genes.
(PDF)

S2 Table Antibody list. (PDF)
