# Supplementary figures and images for: Augmented Growth Hormone Secretion and Stat3 Phosphorylation in an Aryl Hydrocarbon Receptor Interacting Protein (AIP)-Disrupted Somatotroph Cell Line
Source: PLoS One. 2016 Oct 5;11(10):e0164131. doi: 10.1371/journal.pone.0164131 (PMC5051713; doi:10.1371/journal.pone.0164131)

Fig. S1

A

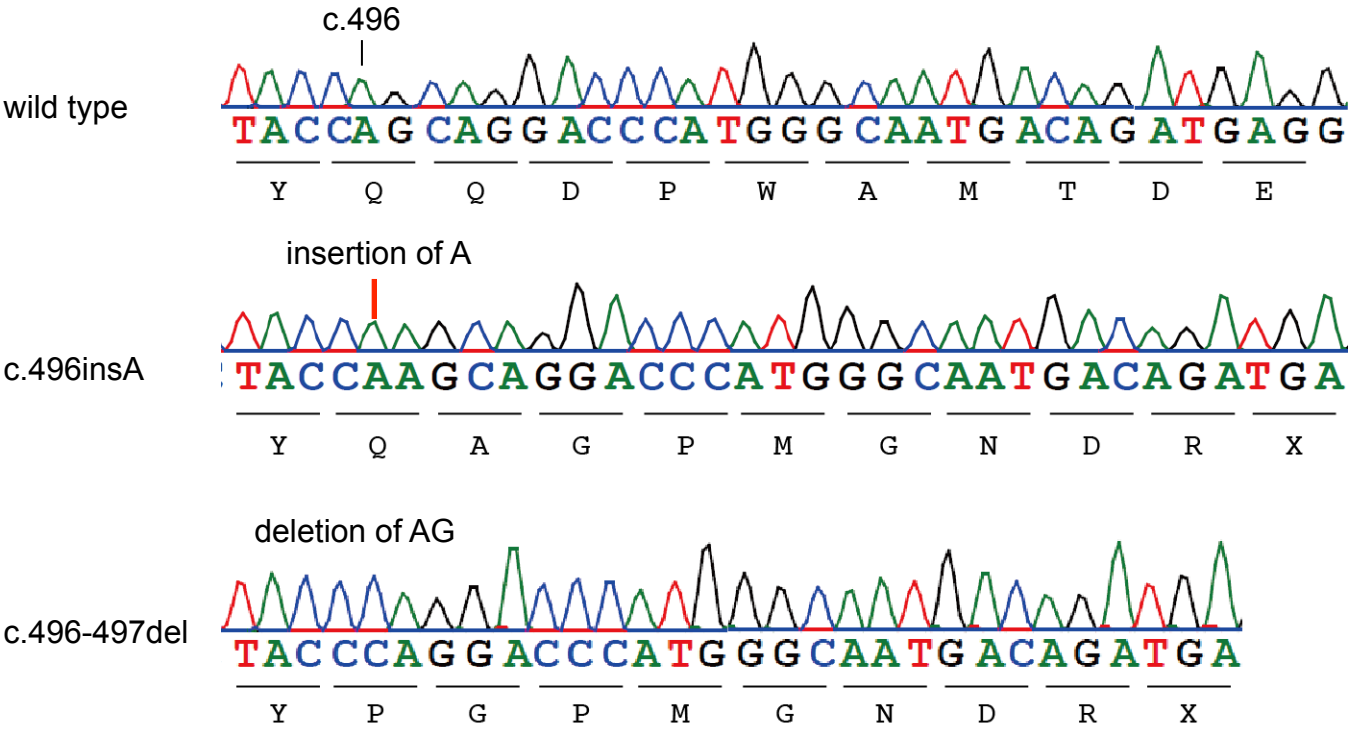

B

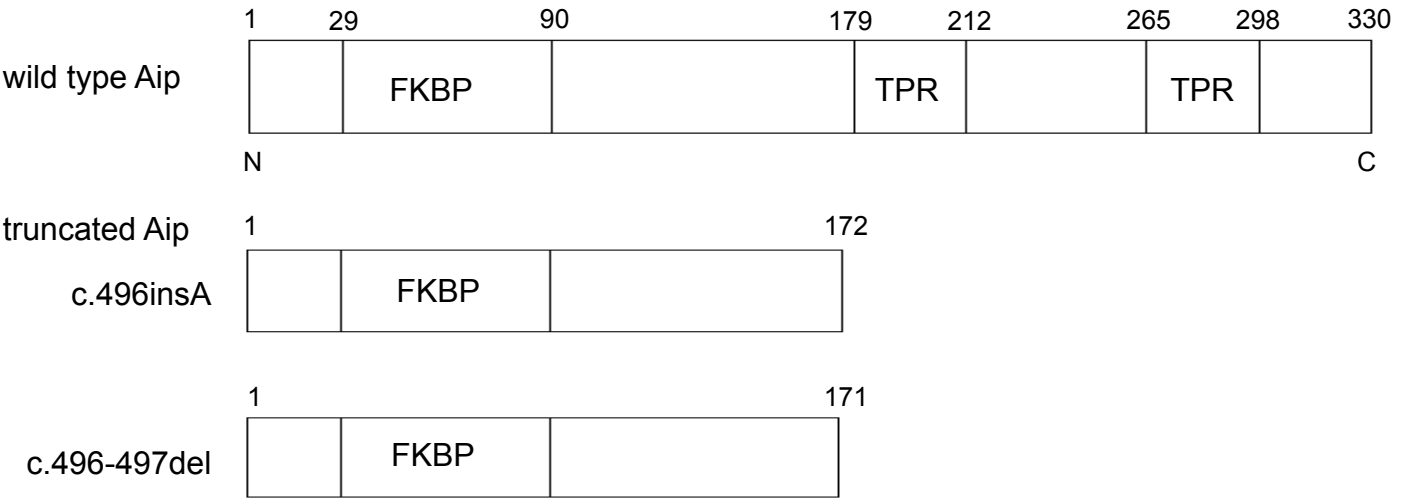

Supplement: S1 Fig — (A) Nucleotide sequence of normal (wild-type) and mutant Aip exon 4 in GH3-FTY cells. GH3-FTY cells contained heterozygous mutants of an adenine insertion at c.496 and a two base deletion at c.496-497, causing premature stop codons at codon 173 (TGA) and codon 172 (TGA), respectively, in both Aip alleles. (B) Schematic of wild-type and mutant Aip structures found in GH3 cells. (PDF) [file pone.0164131.s001.pdf]
