# Supplementary material for: Augmented Growth Hormone Secretion and Stat3 Phosphorylation in an Aryl Hydrocarbon Receptor Interacting Protein (AIP)-Disrupted Somatotroph Cell Line
Source: PLoS One. 2016 Oct 5;11(10):e0164131. doi: 10.1371/journal.pone.0164131 (PMC5051713; doi:10.1371/journal.pone.0164131)
Supplement: S1 Table — Primers 1–6 were used to amplify Aip. Primers 7–13 were used for qPCR to determine the mRNA expression levels of respective genes. (PDF) [file pone.0164131.s002.pdf]

S1 Table. Primer list

#	Target	Sense	Antisense
1	*Aip* exon1	AACCAATCACGGCTCTTTCC	TGACTAGCAGCTGCGCTT
2	*Aip* exon2	ACTGAGATGATGGGATAGAGCC	CCTGAGGATCAGACATAGAGCA
3	*Aip* exon3	CTTCTCTTGTGCCCAGTGAG	GTCAAGGTGGTGATCAGTGAG
4	*Aip* exon4	TGAGAGACAAGTGGTCAGGG	GGACAGTGGAGACGATGAGA
5	*Aip* exon5	GCTGCTGCCAAGTACTACGA	TCAACTGAGGGAGATGTTAAAGC
6	*Aip* exon6	ATGGTGGGCTAGTGCCAGA	CCACTGACTACCAAGCACCTG
7	*growth hormone1 (Gh1)*	CCTTGTCCAGTCTGTTTGCCAAT	CTGGGATGGTCTCTGAGAAGCAG
8	*ploractin (Prl)*	CCAAGATCGTGAGTTTATTGCCAAG	TCCAGGAGTGCACCAAACTGA
9	*aryl-hydrocarbon receptor-interacting protein (Aip)*	CATTGCCTGCCTCAAGAACC	CCGCTTGAAGTAAGCCTTGACA
10	*interleukin 6 receptor (Il6r)*	TCGTTCAGTTCGCACAGGTGTA	CTGGTAAGGATGACCCAAGGATG
11	*somatostatin receptor 2 (Sstr2)*	GAAAGCAGCTACCCGGCAGA	GATTACATAGCGGGCAAGCACAC
12	*pleiomorphic adenoma gene-like 1 (Plagl1)*	CTTTGAAGACTCTGTATTTCCAGCA	GTCTTCAAGGGTTAGGGTACAAATG
13	*actin, beta (Actb)*	GGAGATTACTGCCCTGGCTCCTA	GACTCATCGTACTCCTGCTTGCTG
