# Supplementary material for: Augmented Growth Hormone Secretion and Stat3 Phosphorylation in an Aryl Hydrocarbon Receptor Interacting Protein (AIP)-Disrupted Somatotroph Cell Line
Source: PLoS One. 2016 Oct 5;11(10):e0164131. doi: 10.1371/journal.pone.0164131 (PMC5051713; doi:10.1371/journal.pone.0164131)
Supplement: S2 Table — (PDF) [file pone.0164131.s003.pdf]

Target	Name of Antibody	Manufacturer, catalog #	Species raised in; monoclonal or polyclonal	Dilution used
Aip	AIP/ARA9 antibody (35-2)	Novus, #NB100-127	mouse, monoclonal	1:1000 (WB)
				1:100 (IP)
p-Stat3 (Tyr705)	Phospho-Stat3 (Tyr705) (D3A7)	CST, #9145	rabbit, monoclonal	1:2000 (WB)
				1:100 (IP)
Stat3	Stat3 (D3Z2G)	CST, #12640	rabbit, monoclonal	1:1000 (WB)
				1:100 (IP)
Sstr2	Anti-Somatostatin Receptor 2 antibody [UMB1]	abcam, #ab134152	rabbit, mmonoclonal	1:5000 (WB)
beta-actin	Monoclonal Anti-b-Actin Clone AC-15	SIGMA-ALDRICH, #A5441	mouse, monoclonal	1:5000 (WB)
Rabbit IgG	Anti-rabbit IgG, HRP-linked Antibody	CST, #7074	goat	1:2000 (WB)
Mouse IgG	Anti-mouse IgG, HRP-linked Antibody	CST, #7076	horse	1:2000 (WB)
Gh	Anti-rat Growth Hormone Antibody	R&D, #AF1566	goat	5 μg/mL (IHC-P)
